# UnCoVar: a reproducible and scalable workflow for transparent and robust virus variant calling and lineage assignment using SARS-CoV-2 as an example

**DOI:** 10.1186/s12864-024-10539-0

**Published:** 2024-06-28

**Authors:** Alexander Thomas, Thomas Battenfeld, Ivana Kraiselburd, Olympia Anastasiou, Ulf Dittmer, Ann-Kathrin Dörr, Adrian Dörr, Carina Elsner, Jule Gosch, Vu Thuy Khanh Le-Trilling, Simon Magin, René Scholtysik, Pelin Yilmaz, Mirko Trilling, Lara Schöler, Johannes Köster, Folker Meyer

**Affiliations:** 1 Data Science Research Group, Institute for Artificial Intelligence in Medicine (IKIM), University Hospital of Essen, University of Duisburg-Essen, Essen, Germany; 2 Institute for Virology, University Hospital of Essen, University of Duisburg-Essen, Essen, Germany; 3 Institute for the Research on HIV & AIDS-associated Diseases, University Hospital of Essen, University of Duisburg-Essen, Essen, Germany; 4 Institute of Cell Biology (Cancer Research), University Hospital of Essen, University of Duisburg-Essen, Essen, Germany; 5 Bioinformatics and Computational Oncology, Institute for Artificial Intelligence in Medicine (IKIM), University Hospital of Essen, University of Duisburg-Essen, Essen, Germany; 6 Division of Molecular and Cellular Oncology, Department of Medical Oncology, Harvard Medical School, Boston, MA USA

**Keywords:** SARS-CoV-2, Workflow, Variant calling, Lineage assignment, Next generation sequencing

## Abstract

**Background:**

At a global scale, the SARS-CoV-2 virus did not remain in its initial genotype for a long period of time, with the first global reports of variants of concern (VOCs) in late 2020. Subsequently, genome sequencing has become an indispensable tool for characterizing the ongoing pandemic, particularly for typing SARS-CoV-2 samples obtained from patients or environmental surveillance. For such SARS-CoV-2 typing, various in vitro and in silico workflows exist, yet to date, no systematic cross-platform validation has been reported.

**Results:**

In this work, we present the first comprehensive cross-platform evaluation and validation of in silico SARS-CoV-2 typing workflows. The evaluation relies on a dataset of 54 patient-derived samples sequenced with several different in vitro approaches on all relevant state-of-the-art sequencing platforms. Moreover, we present UnCoVar, a robust, production-grade reproducible SARS-CoV-2 typing workflow that outperforms all other tested approaches in terms of precision and recall.

**Conclusions:**

In many ways, the SARS-CoV-2 pandemic has accelerated the development of techniques and analytical approaches. We believe that this can serve as a blueprint for dealing with future pandemics. Accordingly, UnCoVar is easily generalizable towards other viral pathogens and future pandemics. The fully automated workflow assembles virus genomes from patient samples, identifies existing lineages, and provides high-resolution insights into individual mutations. UnCoVar includes extensive quality control and automatically generates interactive visual reports. UnCoVar is implemented as a Snakemake workflow. The open-source code is available under a BSD 2-clause license at *github.com/IKIM-Essen/uncovar*.

**Supplementary Information:**

The online version contains supplementary material available at 10.1186/s12864-024-10539-0.

## Introduction

Since its first identification, more than 760 million cases of coronavirus disease 2019 (COVID-19) have been reported^1^ since December 2019. The causative pathogen SARS-CoV-2 has affected the lives of billions of people, and researchers found infection or vaccination-induced antibodies in 96% of their subjects in a longitudinal study [1]. High infection rates and continuing uncontrolled transmission led to the emergence and spread of viral lineages carrying fitness-enhancing mutations [2–11], while controlling transmission and vaccination promoted the evolution of immune-evasive alterations in the viral genome [12]. Due to their relatively high transmissibility [3, 5, 10, 13, 14], such variants of concern (VOCs) carrying mutations beneficial for the virus have replaced the wild type [3, 15, 16], making whole-genome sequencing with next-generation sequencing (NGS) approaches instrumental for assessing the genomic diversity of the virus in patients.

Previous work has focused on the reconstruction of virus genomes [17–22] and the surveillance of SARS-CoV-2 genomes [23–25]; however, limited attention has been given to reproducibility and portability. In addition, no comprehensive multiplatform benchmark dataset from various protocols and sequencing instruments, including Sanger sequences as ground truth for assessing SARS-CoV-2-related workflows, has been devised thus far. In this work, we present both benchmark dataset and UnCoVar, a reproducible, transparent, and scalable analysis workflow that accepts sequencing products from various protocols. UnCoVar has been extensively optimized for SARS-CoV-2 in routine clinical application and environmental surveillance during the pandemic while being straightforwardly adaptable to future outbreaks of other viruses.

## Methods

### The UnCoVar workflow

UnCoVar is a Snakemake [26] workflow for virus analysis that provides full in silico reproducibility, diagnostic transparency, uncertainty awareness, and extensive interactive graphical exploration of results.

UnCoVar is publicly available at https://github.com/IKIM-Essen/uncovar under the BSD-2-clause license. Detailed information on the software tools and libraries used in UnCoVar and its usage are available at https://ikim-essen.github.io/uncovar.

**Fig. 1:**
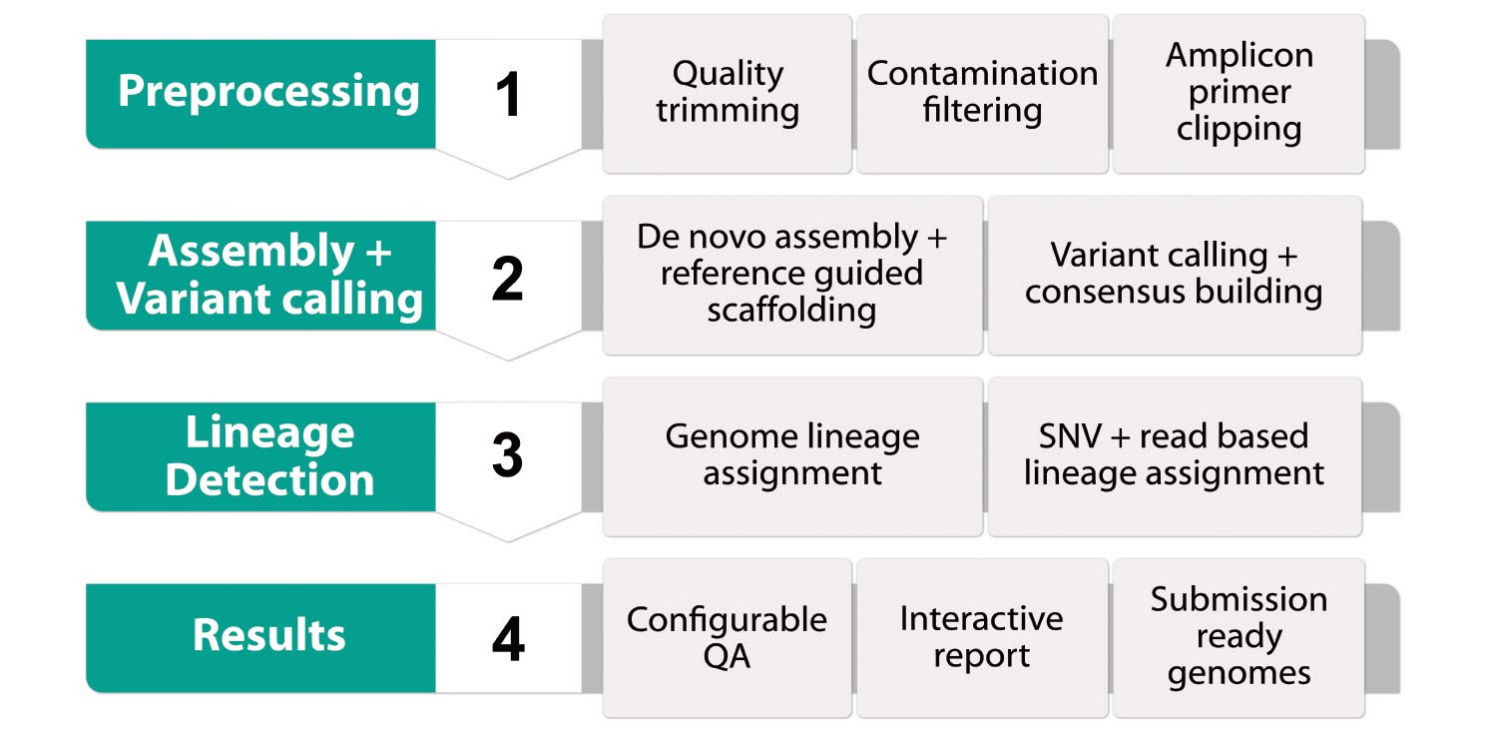
Outline of UnCoVar. The individual steps of the workflow can be summarized as follows: preprocessing, assembly and variant calling, lineage detection and result generation

UnCoVar consists of four main modules: (i) preprocessing and quality control of raw sequence data, (ii) de novo assembly, reference-guided scaffolding, variant calling and consensus building, (iii) lineage detection, and (iv) aggregation of results and report generation (Fig. 1). Instructions on installation, deployment, configuration and execution as well as a detailed description of the tool chain can be found in the online documentation.

The approaches used for quality control, preprocessing, assembly and lineage detection are described in Table 1. We highlight that only the lineage detection tool Pangolin is SARS-CoV-2 specific (specificity tested against Non-SARS-CoV-2 Corona viruses; Appendix Chap. 5), and the pipeline can easily be adapted to other viral pathogens by registering the respective reference genomes and using Kallisto [27] instead of Pangolin [28] for lineage detection. Moreover, we expect Pangolin (or a successor) to be adapted in the case of future non-SARS-CoV-2 pandemics. With this amount of flexibility, UnCoVar serves the concept for a Disease X [29] analysis tool, a yet unknown pathogen with the potential for an endemic or pandemic outbreak.

**Table 1:** Tools used in UnCoVar depending on the type of input data (Illumina short reads or Nanopore long reads)

Stage	Step	Tool Illumina		Tool Nanopore	SARS-CoV-2 specific
Preprocessing	primer clipping	BAMClipper [30]		NoTrAmp [31]	no
	quality clipping	fastp [32]			
	contamination removal	Kraken2 [33]			
	Denoising	***^a^		Canu [34], Medaka [35]	
Assembly	Assembly^b^	MEGAHIT [36], metaSPAdes [37]			
	scaffolding	RaGOO [38]			
	polishing	BCFtools consensus [39]	Medaka consensus		
Variant calling	SNV calling	freeBayes [40], DELLY [41]		Medaka variant, Longshot [42]	
	SNV validation	Varlociraptor [43]			
Lineage detection	read based lineage assignment	Kallisto [27]			
	lineage call	Pangolin [28]			yes

^a^No denoising is performed for Illumina reads. Instead, assembly products are polished with uncertainty-aware variant calls from Varlociraptor

^b^Besides the default assembly options, Trinity, Velvet, MEGAHIT-meta large/sensitive and corona-/rnaviral-/ standard SPAdes are available for use

UnCoVar is adjustable via a thoroughly documented configuration file. It supports whole-genome shotgun and amplicon-based sequencing from Illumina and Nanopore sequencing and has been extensively tested with data from both sequencing methods from a clinical dataset. In the following, we provide methodological details of the major functionalities of UnCoVar.

### Variant calling

UnCoVar employs technology-specific variant callers (short reads: freeBayes [40] for small variants and DELLY [41] for structural variants; long reads: Medaka variant [35] for small variants and Longshot [42] for structural variants) to obtain a list of candidate variants for each investigated sample. The candidate variants are subsequently given to the generic variant classification functionality of Varlociraptor^2^ [43].

### Complementary genome reconstruction methods

A variety of assemblers have been compared and two default assembly options have been selected according to each library preparation method (MEGAHIT [36] for shotgun, metaSPAdes [37] for amplicon derived samples) based on the best performance (Appendix Chap. 4 and Figures A2 + A3). All alternative assembly tools tested (Trinity [44], Velvet [45], MEGAHIT-meta large/sensitive and corona- [46] /rnaviral-/ standard SPAdes [47]), are available and can be used via selecting them in UnCoVar’s config file. The pipeline offers two approaches for determining the genomic sequence of a given virus sample. The first (and preferred) approach uses de novo assembly, followed by reference-guided scaffolding. Then, reads are mapped against the obtained assembly using BWA-MEM [48], and variants are called with Varlociraptor (see section Variant calling). Variants for which the subclonal-major, subclonal-high and clonal events summed to at least a probability of 0.95 were used to polish the assembly. Low-quality loci (those with low read depth and ambiguous basecalls) are masked by N or IUPAC codes^3^

The second approach maps reads against the primary reference genome of the investigated virus and applies the above polishing approach to the reference genome, including the masking of uncovered regions.

### Lineage assignment

Lineage calling approaches fall into two distinct classes: those requiring an almost fully reconstructed genome sequence [49] and those using raw sequencing products in the form of reads, without the necessity of sequence assembly [50–55].

UnCoVar offers three approaches for assigning lineages to samples. First, based on the obtained genome reconstruction, in the case of SARS-CoV-2, UnCoVar utilizes the machine learning driven method Pangolin [28] to assign a lineage.

Second, it employs Kallisto [27] to quantify the numbers of reads originating from given lineage reference sequences, and subsequently calculates their fraction among the total amount of mappable reads. This approach has the advantage of being able to detect lineage mixtures within a single sample, which can allow the detection of mixed infections or the assessment of wastewater samples.

To account for the rapid evolution of SARS-CoV-2, UnCoVar offers a comparison between the investigated sample and the most similar lineages at the level of individual variants. The pipeline obtains the catalog of all known amino acid and noncoding alterations of variants/lineages of concern (VOCs) available on covariants.org. Amino acid alterations are back-translated into all potential causing multiple nucleotide variants (MNVs). The resulting set of candidate variants is called using Varlociraptor (see section Variant calling, leveraging Varlociraptor’s functionality to classify any set of candidate variants). To determine the degree of similarity between the sample and the VOCs, we performed the following scoring. Let *n* be the total number of variants and *m* be the number of VOCs. Let *X* be a binary matrix that relates variants with VOCs, namely, *X*_*i,j*_ = 1 if and only if variant *i* is in *VOC*_*j*_, with *i* = 1,…,*n* and *j* = 1,…,*m*. Let *θi* be the latent allele frequency of variant *i* in the given sample and ${{\hat \theta }_i}$ be the maximum a posteriori estimate of *θi* as provided by Varlociraptor. Let ${p_i} = Pr\left( {{\theta _i}\, > \,0\,|\,D} \right)$ be the posterior probability that the variant *i* is present in the sample (i.e., the probability that its latent allele frequency is greater than zero, given the data *D*). Then, the similarity of a given sample to *VOC*_*j*_ can be calculated as the Jaccard-like similarity score:

$${{\sum\limits_{i = 1}^n {{p_i} \cdot \,{{\hat \theta }_i} \cdot {X_{i,j}} + \left( {1 - {p_i}} \right) \cdot \left( {1 - {X_{i,i}}} \right)} } \over n}$$

The better the variant pattern of a VOC is matched (present true positive plus absent true negative mutations), the closer the similarity score is to one. In contrast, if the variant pattern tends toward being the opposite of a VOC, the corresponding score tends toward zero. A low maximum a posteriori VAF estimate or a weaker probability lowers the summand for a specific variant $i$, such that the overall score decreases.

In this way, UnCoVar is capable of assigning lineage similarities without a fully reconstructed genome, which is especially useful when the analyzed samples are derived from patients with low-level viremia or from environmental sewage water samples, where the abundance of viral RNAs is low and potentially contains several different lineages. UnCoVar reports the top 10 VOC lineages found based on the calculated Jaccard-like similarity of all present and absent mutations included in the VOC database.

### Graphical report

**Fig. 2:**
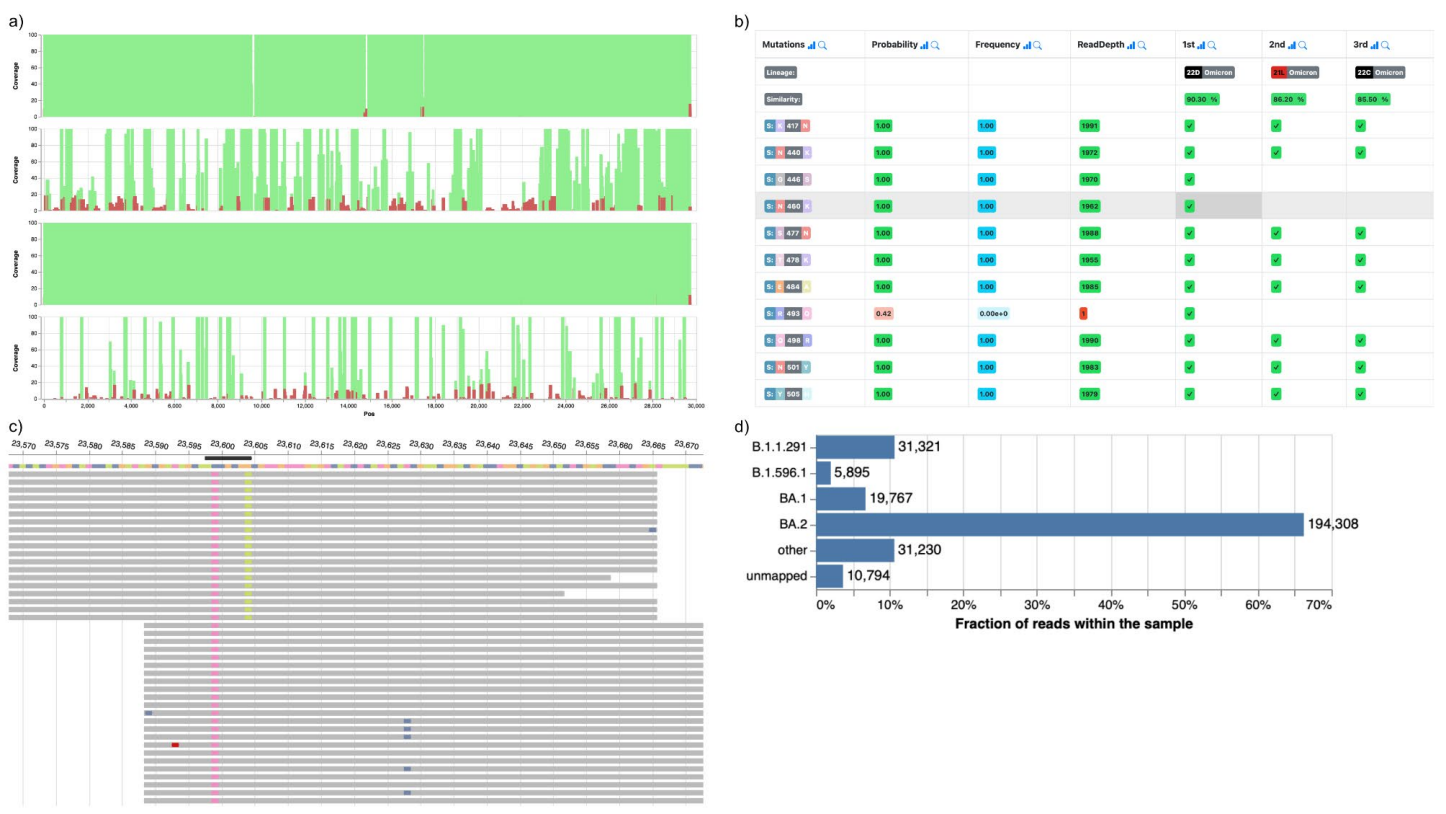
Four different example elements of the results generated by UnCoVar: **(a)** The genome coverage of the aligned reads, visualized for multiple samples, **(b)** evaluation of known protein alterations from VOCs for one sample, **(c)** a pileup of reads at the position of one protein alteration. The mutations observed for multiple reads (gray bars) for a single sample, here in the S gene, **(d)** The lineage assignments inferred for single reads for one sample. Unmapped reads can be attributed to low sequence quality and variation beyond the considered lineages

UnCoVar’s high-level interactive graphical reporting interface allows noncomputational scientists to navigate the details of the analysis and results. The user interface provides an accurate picture of uncertainties in the data. The Snakemake-generated report is portable and maintenance-free since it does not require a running and constantly maintained database or web service. It can be easily archived, distributed via email, a static web server, or any file-sharing platform and solely requires an HTML5^4^ compliant web browser to be viewed (see Fig. 2). A detailed overview of all included results can be found on the GitHub pages of UnCoVar (https://ikim-essen.github.io/uncovar/*).*

### The benchmark dataset

We present a benchmark dataset of 54 clinical SARS-CoV-2 patient samples. These 54 samples were obtained from SARS-CoV-2 qPCR-positive patients at the University Hospital Essen, Germany, covering the period from February to September 2021^5^. For all the samples, cDNA synthesis was conducted using the LunaScript^®^ RT SuperMix kit (New England Biolabs, USA). Subsequently, all samples underwent Sanger sequencing for a portion of the genome region encoding the S protein as well as two separate procedures for the targeted sequencing of complete SARS-CoV-2 genomes by tiled amplicons. First, a sequencing library was prepared according to the LoCost nCoV-2019 sequencing protocol (Quick) using the ARTICv3 primer panel (Integrated DNA Technologies, Coralville, Iowa) for loading onto an R9.4.1 Flow Cell and subsequent sequencing on a GridION device (Oxford Nanopore Technologies, Oxford, United Kingdom). Second, library preparation was performed using the EasySeq™ SARS-CoV-2 WGS kit (NimaGen, Nijmegen, The Netherlands), followed by sequencing on an Illumina MiSeq sequencing platform (Illumina Inc., San Diego, CA, USA). For a subset of 32 samples, library preparation was additionally performed using the Illumina COVIDSeq Assay Kit (Illumina Inc., San Diego, CA, USA) followed by sequencing on an Illumina MiSeq platform. The approaches of the Illumina and NimaGen library preparation kits are similar, except that the EasySeq kit combines cDNA amplification and index PCR in a single reaction. Since the dataset was collected in the middle of the SARS-CoV-2 pandemic, we carefully curated the matching between the different technologies to rule out human errors. As a result, we discarded 22 Illumina samples due to potential sample swaps. The data generated by all different approaches were then analyzed and used to benchmark precision and recall across different sequencing platforms in terms of calling individual mutations, virus lineages, and sequence assemblies. The raw NGS benchmarking data is available for download at https://www.ebi.ac.uk/ena/browser/view/PRJEB73579.

## Results

Using the presented benchmark dataset, we analyzed the performance of UnCoVar and other state-of-the-art analysis pipelines in terms of precision, recall and runtime. Furthermore, we investigated the required number and length of reads needed for accurate lineage assignment in UnCoVar and examined the time required to execute the workflow. Finally, we compared UnCoVar against other available pipelines using the benchmark dataset described above.

### Benchmarking

To assess the different sequencing protocols, the preprocessed reads (see Methods) were aligned to the primary SARS-CoV-2 reference genome from Wuhan (NC_045512.2 or MN908947), and variants were called using UnCoVar. Only variants with a posterior probability ≥ 0.95 for presence according to Varlociraptor were considered, thereby controlling the local false discovery rate in a Bayesian sense at 0.05. The observed variants were compared with those found in the Sanger sequence in the corresponding region. Variants outside of the Sanger sequenced region were omitted. If a variant also occurred in the Sanger sequencing, it was considered a true positive; if not, it was considered a false positive (assuming that Sanger sequencing has the highest possible accuracy). Sanger-based variants that did not occur in the investigated sample were considered false negatives. Let TP, FP, and FN be the respective numbers of true positives, false positives, and false negatives across all samples. We defined precision as the fraction TP/(TP + FP) of true positives among all predicted variants and recall as the fraction TP/(TP + FN) of true positives among all variants in the Sanger sequences. Obviously, the recall can drop with decreasing sequencing depth. More details on the sequencing depth necessary for proper lineage assignment can be found in Appendix Chap. 6. In general, we can conclude, that Pangolin and Kallisto perform equally well in case of nearly complete assemblies while the lineage prediction accuracy of Pangolin decreases with the of completeness of the assembly (Appendix Chap. 6, Figure A4).

As shown in Table 2, the UnCoVar pipeline achieves outstanding precision and recall when using Sanger sequencing data as a true positive gold standard across three different in vitro technologies. Importantly, the precision always stays above the expectation of 0.95 defined by the controlled FDR of 0.05, indicating that the statistical model of Varlociraptor used in UnCoVar manages to properly assess the uncertainty in the data.

**Table 2:** UnCoVars precision/recall of observed variants per sequencing kit vs. observed variants of Sanger sequencing. *Reduced number of samples for the Illumina kit due to potential sample swaps; see methods

	Sanger	Artic/ONT	Illumina	NimaGen
Variants	160/85*	160	83*	161
Precision	-	1.0	1.0	0.97
Recall	-	1.0	0.98	0.98

### Comparison with other pipelines

We compared UnCoVar against other available state-of-the-art pipelines using the above-mentioned benchmark dataset (Table 3).

**Table 3:** Computing time and precision/recall comparison of the identified variants between UnCoVar and three other state-of-the-art pipelines. Computing time is given as the median computing time per sample when running all considered benchmark samples and performing only the variant callings. *Reduced number of samples for Illumina kit due to potential sample swaps (Nimagen/ONT = 54 samples; Illumina = 32 samples)

Kit + Sequencer	Pipeline	Precision	Recall	Computing time (h: mm: ss)
Illumina/Illumina*	UnCoVar	1.0	0.98	0:03:44
	Nf-core-viralrecon [53]	0.98	0.99	0:03:46
	V-Pipe [56]	0.66	0.95	1:15:41
	CoVPipe [57]	0.92	0.99	0:06:41
NimaGen/Illumina	UnCoVar	0.97	0.98	0:06:48
	Nf-core-viralrecon	0.99	0.76	0:05:21
	V-Pipe	0.9	0.33	1:12:46
	CoVPipe	0.78	0.87	0:04:18
Artic/ONT	UnCoVar	1.0	1.0	0:02:02
	Artic-medaka [58]	0.98	1.0	0:06:03

All but two of the pipelines achieved a precision and recall above 0.90. This represents a satisfactory result both for the software developers and for clinicians using the results from these pipelines. UnCoVar was the only pipeline that consistently achieved at least 0.97 for both precision and recall across the different in vitro platforms used. When measuring and comparing the execution time to produce variant callings between all considered workflows (average of the individual processing time for all 54 samples), UnCoVar was the fastest for one and close to the compared other pipelines in the other Illumina in vitro approach. A more detailed view of the run times of UnCoVar with differing sequencing depths can be found in Appendix Chap. 2. For Oxford Nanopore data, UnCoVar is one of two pipelines capable of processing such samples without errors at the time of writing, with perfect precision and recall rates and the quickest average execution time measured for all 54 samples. We note that we cannot guarantee overall correct usage of the other software products and executed the compared workflows based on the available documentation. Any bugs that occurred were reported to the original authors. An overview of the tested pipelines and reasons for exclusion can be found in the appendix (Appendix Chap. 7, Table A1).

We posit that the presence of a vendor- and platform-agnostic gold standard for NGS data supported by non-NGS data will enable other groups to use the data for benchmarking their approaches.

## Discussion

We present UnCoVar, a fully automated, reproducible workflow for analyzing viral pathogen sequencing data. In addition, we present a thoroughly investigated gold standard benchmark dataset of 54 SARS-CoV-2 samples sequenced with multiple technologies. Using this dataset, we show that UnCoVar outperforms all other available analysis pipelines in terms of recall and precision. UnCoVar thereby manages to accurately control the false discovery rate using Varlociraptor [43].

By using a combination of Snakemake [26], Conda/Mamba, and Snakedeploy, the workflow is portable, reproducible, transparent, and adaptable to any viral pathogen. A combination of different state-of-the-art tools delivers a robust analysis that accepts sequencing products from a range of different instruments and protocols as input.

During the SARS-CoV-2 pandemic, rapid viral mutations played a major role in increasing infection rates [12, 59–61]. While other approaches [56, 57] commonly use only one strategy for crucial steps in the analysis (e.g., de novo assembly or SNV-based consensus building), UnCoVar provides complementary functions for assembly, variant calling, genome reconstruction, and lineage identification. With the strength of using Varlociraptor and its powerful features for the probabilistic re-evaluation of identified mutations, we integrated a unique addition to conventional variant calling methods, as confident identification of SNVs and other mutations played a crucial role in pandemic surveillance. The widely used tool Pangolin for SARS-CoV-2 lineage assignment depends on accurate genome assembly, which UnCoVar achieves by automated SNV-based consensus building, integrated quality assurance and postprocessing of reconstructed genomes. While this is commonly achieved when sequencing patient samples, a lack of full-genome amplification and sequencing and therefore, incomplete genome assembly often occurs in the case of analyzing environmental – for example, wastewater – samples. Furthermore, evaluating known SARS-CoV-2 protein alterations and not being dependent on a fully reconstructed genome allows us to identify the occurrence of new virus variants through the exclusivity of specific mutations. By providing all these “belts and suspenders”, UnCoVar is a versatile all-in-one pipeline with considerable potential, not only for analyzing SARS-CoV-2 samples.

Future work will entail the potential addition of BUSCO [62] for assembly quality assessment. Moreover, we will investigate the use of pangenome references [63] for further improving contamination detection and reducing reference bias in read alignment.

We will work on updating the benchmark dataset with additional SARS-CoV-2 variants and attempt to include other sequencing platforms. As we continue to analyze patient-derived samples from our institution, we will maintain the SARS-CoV-2 analysis and include additional viral pathogens (RSV, influenza A and B) for analysis with UnCoVar. UnCoVar was efficiently employed for the characterization of SARS-CoV-2 variants from wastewater samples [64], and a prototypical module of UnCoVar was employed in a SARS-CoV-2 surveillance project at neighborhoods and city scales in the metropolitan Ruhr area of Germany (Thomas et al., in preparation).

### Availability and requirements

Project name: UnCoVar.

Project home page: *github.com/IKIM-Essen/uncovar*.

Operating system(s): platform independent.

Programming language: Python.

Other requirements: Conda, Snakemake 6.9. or higher.

License: BSD-2-Clause License.

Any restrictions to use by non-academics: None.

### Electronic supplementary material

Below is the link to the electronic supplementary material.

Supplementary Material 1

## Data Availability

Sequence data that support the findings of this study have been deposited in the European Nucleotide Archive with the primary accession code PRJEB73579.

## Footnotes

1 https://www.who.int/news-room/fact-sheets/detail/coronavirus-disease-(covid-19).

2 Using Varlociraptor’s variant calling grammar: https://varlociraptor.github.io/docs/calling/#generic-variant-calling.

3 In line with criteria defined by the Robert-Koch-Institute, Germany: https://www.rki.de/DE/Content/InfAZ/N/Neuartiges_Coronavirus/DESH/Qualitaetskriterien.pdf? __blob=publicationFile

4 http://www.w3.org/TR/html5.

5 ethics vote #20-9512-BO.

## Abbreviations

COVID-19: Coronavirus Disease 2019
NGS: Next-Generation Sequencing
SARS-CoV-2: Severe Acute Respiratory Syndrome Coronavirus 2
VOCs: Variants of Concern
SNVs: Single Nucleotide Variants
