# Supplementary material for: UnCoVar: a reproducible and scalable workflow for transparent and robust virus variant calling and lineage assignment using SARS-CoV-2 as an example
Source: BMC Genomics. 2024 Jun 28;25:647. doi: 10.1186/s12864-024-10539-0 (PMC11214259; doi:10.1186/s12864-024-10539-0)
Supplement: Supplementary file 1 — Supplementary Material 1 [file 12864_2024_10539_MOESM1_ESM.docx]

UnCoVar: A Reproducible and Scalable Workflow for Transparent and Robust Virus Variant Calling and Lineage Assignment using SARS-CoV-2 as an Example

Alexander Thomas^1+^, Thomas Battenfeld^1+^, Ivana Kraiselburd^1^, Olympia Anastasiou^2^, Ulf Dittmer^2^, Ann-Kathrin Dörr^1^, Adrian Dörr^1^, Carina Elsner^2^, Jule Gosch^1^, Vu Thuy Khanh Le-Trilling^2,3^, Simon Magin^1^, René Scholtysik^3^, Pelin Yilmaz^1^, Mirko Trilling^2,3^, Lara Schöler^2,3^, Johannes Köster^5x^, Folker Meyer*^1x^

1 Data Science Research Group, Institute for Artificial Intelligence in Medicine (IKIM), University Hospital of Essen, University of Duisburg-Essen, Essen, Germany

2 Institute for Virology, University Hospital of Essen, University of Duisburg-Essen, Essen, Germany

3 Institute for the Research on HIV & AIDS-associated Diseases, University Hospital of Essen, University of Duisburg-Essen, Essen, Germany

4 Institute of Cell Biology (Cancer Research), University Hospital of Essen, University of Duisburg-Essen, Essen, Germany

5 Bioinformatics and Computational Oncology, Institute for Artificial Intelligence in Medicine (IKIM), University Hospital of Essen, University of Duisburg-Essen, Essen, Germany

+ Shared first authors
x Shared last authors

* Corresponding author:

Prof. Dr. Folker Meyer

Institute for Artificial Intelligence in Medicine (IKIM), University Hospital Essen

Hufelandstr. 55, 45147 Essen, Germany

Email: [folker.meyer@uk-essen.de](mailto:folker.meyer@uk-essen.de)

## Supplementary Material

## Appendix 1: Pipeline implementation details

As input, UnCoVar requires short-read sequencing data in paired-end FASTQ format (e.g., produced by the Illumina platform) or long-read data in single-end FASTQ format (e.g., produced by the Oxford Nanopore platform), a sample sheet containing information about the reads, and a (predefined) configuration file. Several different Illumina platform protocols for SARS-CoV-2 sequencing exist. The spectrum ranges from shotgun, e.g., [1], to multiplex tiling PCR-based approaches, e.g., [2-5], each posing specific challenges for downstream processing.

If the raw data was generated via an amplicon procedure, e.g., multiplex tiling PCR sequencing, an additional alignment file containing primer pairs with genomic coordinates can be provided for gene-specific primer sequence clipping.

UnCoVar uses BWA-MEM [6] for read alignment and minimap2 [7] for contig and genome alignment. For preprocessing and quality control of the raw data, UnCoVar uses fastp [8], and BAMclipper [9] (in case of amplicon reads). First, reads with an average Phred quality less than 20 and a length less than 30 base pairs are excluded. The thresholds can be modified via the configuration file. Next, adapter trimming is performed. The adapter sequences to be trimmed can also be specified in the configuration file. Finally, any host contamination is removed by aligning the reads against a combined reference of the human (defaults to GRCh38.p13) and the SARS-CoV-2 reference genome (defaults to NC_045512.2). These references are adjustable via the configuration file. All reads mapping the human reference genome are discarded, and only sequences that are either unaligned or aligned to the SARS-CoV-2 reference genome are considered in subsequent steps. For reads generated by an amplicon procedure, gene-specific primer sequences are removed by hard-clipping.

Preprocessed and quality-controlled reads are de novo assembled using MEGAHIT [10] or, in the case of amplicon data, by metaSPAdes [11]. To improve the assembly results, the resulting contigs are subsequently scaffolded against the SARS-CoV-2 reference genome sequence using raGOO [12].

To detect candidate single and multiple nucleotide variants (SNVs, MNVs) as well as small insertions and deletions (indels), Freebayes [13] is employed, whereas for structural variants, Delly [14] is used. Once candidates are detected, Varlociraptor [15] is utilized to evaluate the candidates with a unified statistical model. Artifacts are separated from genuine variants via a false discovery rate control.

This variant calling approach is used in three different ways. First, we use it to polish de novo assemblies (thereby mapping and comparing reads against the assembly instead of the SARS-CoV-2 wild-type reference genome) by employing BCFtools consensus [16] (for Illumina data) and Medaka variants (for Nanopore data) to apply variants of sufficient allelic fraction to the assembled virus genome. Likewise, we mask unclear loci (using configurable thresholds for coverage and minimum major allele fraction) with the corresponding IUPAC codes. Second, we use the called variants to alter the SARS-CoV-2 wild-type reference genome, thereby generating a reference-guided consensus sequence. This can serve as a fallback solution in cases where the de novo assembly fails (e.g., due to missing coverage). However, de novo assembly should be preferred, as pseudoassembly naturally introduces bias toward the reference genome. Third, variant calling is used to report individual variants for detailed inspection.

SARS-CoV-2 lineage assignment of the assembly is done using Pangolin [17], which employs the dynamic nomenclature for SARS-CoV-2 lineages suggested by Rambaut *et al*. [18]. The underlying Pangolin database, used by pangoLEARN, is updated automatically for each run of the workflow to provide the most up-to-date nomenclature. The most current version of Pangolin should provide the best possible result. However, if the user wants to reproduce past analyses, it is still possible to downgrade the version of Pangolin and Pangolin-data by adjusting the respective Conda environment. Additionally, Kallisto [19] quantifies abundances of sequences belonging to SARS-CoV-2 lineages (which can be downloaded and prepared via GISAID) using preprocessed and quality-controlled short reads. These quantifications can be used to verify the predictions of Pangolin. In addition, this approach enables the detection of mixed infections with several lineages.

UnCoVar provides a comprehensive and interactive interface that aggregates data from various steps of the workflow into six main sections. The first section displays plots of lineage assignments across all patient samples over time (all plots are produced using Altair [20, 21]. A summary table is provided, which contains read counts for the raw, trimmed, and filtered reads, as well as the length of the longest initially assembled contigs. Additionally, the lengths of the scaffolded contigs and the reference-guided pseudoassembled sequences are displayed. An overview of contamination per sample based on the raw reads is provided (determined by Kraken2 [22], using the MiniKraken2 v2 8GB database). The assigned SARS-CoV-2 lineages and variant calls with their corresponding allele frequencies are displayed.

The second section of the report contains detailed information on variant calls, explorable using an interactive graphical interface. For each gene, the variants, impact, consequence, and allele frequency can be investigated. In addition, all the statistical evidence from Varlociraptor’s model and individual read alignments are visualized.

Section 3, “Sequencing Details”, provides a sanity check of the sequencing run and the assemblies. In addition to plots displaying the read coverage of the SARS-CoV-2 reference genome and the assembled sequence, MultiQC [23] is used to aggregate information throughout various stages of the workflow. For example, the raw short-read data is visualized by parsing the output from FastQC [24] and Kraken2. Several assembly metrics can be examined more closely by investigating the output from QUAST [25]. The necessary alignments of the contigs to the SARS-CoV-2 reference are performed by minimap2 [7].

The next two sections of the report focus on data access and provision. In addition to the final polished assembled sequences, variant call files are available for download. This data can be examined further, e.g., by uploading it to the UCSC SARS-CoV-2 Genome Browser [26].

The last section aggregates all sequences with at least 90% identity to the SARS-CoV-2 wild-type reference genome and whose proportion of Ns in the reconstructed genome is less than or equal to 5%. This section is aimed explicitly at health institutions (such as the German Robert Koch Institute), which require high-quality genome data, e.g., for molecular surveillance.

## Appendix 2: Runtime

Runtime tests were carried out on an Intel Xeon Silver 4216 CPU (64 cores, 2.1 GHz) with 191 GB of memory. When using UnCoVar for the first time, various environments are installed, databases are downloaded, and indexes are created. This fully automated initial setup took approximately one hour (57 min ± 4.2 min) and only needed to be carried out once. To evaluate the runtime of UnCoVar, four datasets with 20 samples per set were simulated. The sizes of the datasets were varied by generating different numbers of reads per sample. These sizes correspond to the 25%, 50%, 75% and 100% quantiles of numbers of reads per sample seen in historical clinical data. UnCoVar was executed ten times on each dataset, and the runtime for running the complete workflow (excluding time for initial setup) was measured (Figure A1).

The median processing times of UnCoVar were as follows:

- 18 minutes for a sample with 130,000 reads per paired-end FASTQ file,
- 24 minutes for a sample with 300,000 reads,
- 46 minutes for a sample with 540,000 reads, and
- 2 hours and 2 minutes for a sample with 5,000,000 reads.

Since UnCoVar is based on Snakemake, computing steps are automatically parallelized based on user specified resource constraints and the utilized underlying computing infrastructure (workstation, compute server, cluster, cloud, see [27]).


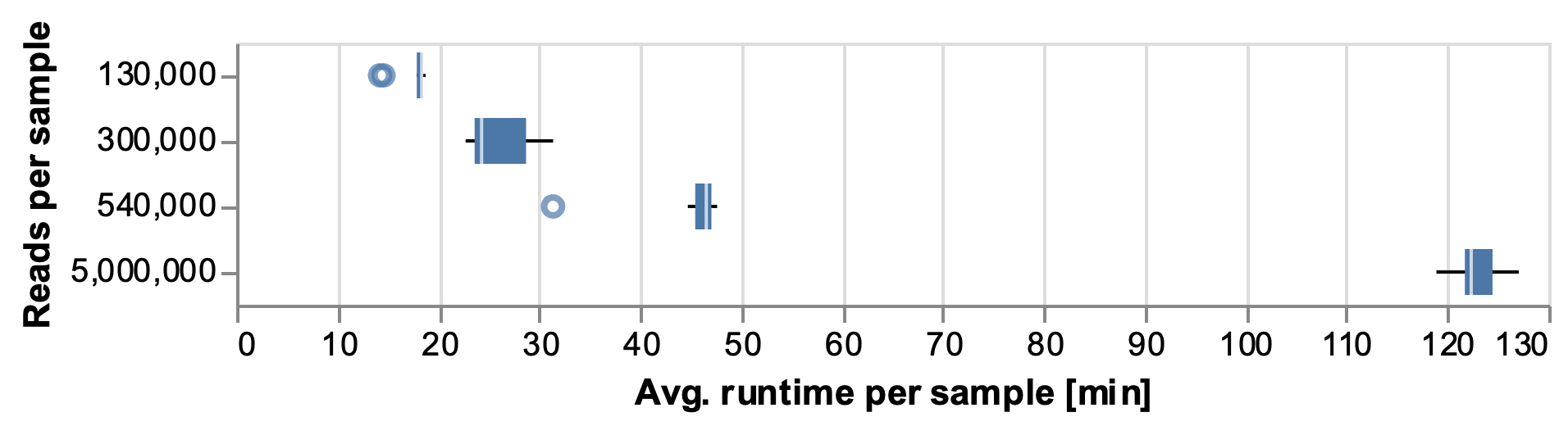


Figure A1: Distribution of UnCoVar’s average runtime per sample depending on the input size of the sample.

## Appendix 3: Additional information about the Benchmark Dataset

The clinical dataset was compiled from available SARS-CoV-2 qPCR-positive patient samples from the University Hospital of Essen covering the period from February to September 2021. All samples were processed with NGS on two different sequencing instruments for whole-genome reconstruction and Sanger sequencing for specific regions of interest within the virus spike protein.

Illumina MiSeq instrument:

- EasySeq RC-PCR SARS CoV-2 Whole Genome Sequencing Kit, NimaGen, Netherlands
- Illumina COVIDSeq Assay Kit, Illumina Inc., USA

Oxford Nanopore GridION instrument:

- ARTIC nCoV-2019 Amplicon Panel v3, Integrated DNA Technologies, USA

The reference dataset can be downloaded from the European Nucleotide Archive: https://www.ebi.ac.uk/ena/browser/view/PRJEB73579

## Appendix 4: Comparison of Assemblers

Using the benchmark dataset, which was additionally prepared with shotgun sequencing (data not shown in the main results), nine different short-read assembly options from four assemblers were tested to determine the most suitable options for SARS-CoV-2 genome assembly. The metrics used for assessment were the N50, the length of the longest contigs after assembly, the length of the sequence after reference-guided scaffolding and the concordance of the contigs with the SARS-CoV-2 reference genome (Figure A2).


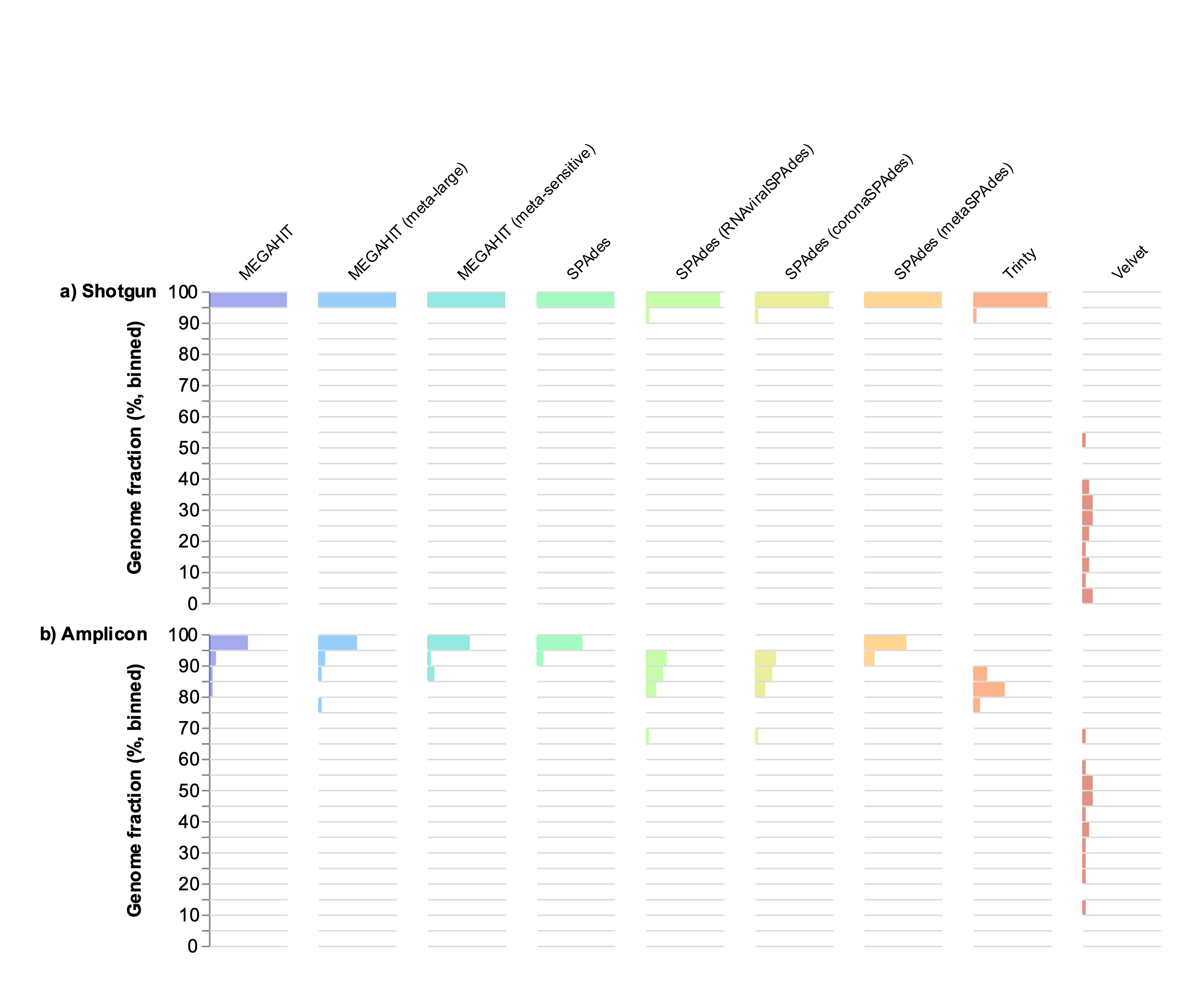


Figure A2: Histograms of SARS-CoV-2 reference genome fractions represented in the contigs generated with the different tested assemblers (evaluated with QUAST; bins=20). Samples of the clinical SARS-CoV-2 benchmark dataset processed via a) shotgun sequencing and b) multiplex tiling PCR (Amplicon). Colors represent different assemblers. The desired result for a high-quality assembly is 100 percent representation of the reference genome in the assembled contigs. Mismatching contigs result in the abundance of the bins with lower percentage.

For shotgun sequencing, MEGAHIT, with default settings, produced the highest N50 values and assembled the largest contigs. The resulting scaffolded sequences showed the smallest deviation from the 30 kb length of the reference sequence (Figure 2a). Additionally, the reads of all clinical samples covered at least 95% of the reference genome (Figure A2a).

For multiplex tiling PCR data, SPAdes and metaSPAdes were able to assemble the longest initial contigs (Figure A2b). With slightly longer scaffolded sequences and at least 90% (Figure A2b) reference genome fraction, metaSPAdes was chosen for amplicon-based data. Both selections are in accordance with the findings of Islam *et al*. [(Islam et al., 2021)](https://web.endnote.com/citations/eyJkaXNwbGF5VGV4dCI6IihJc2xhbSBldCBhbC4sIDIwMjEpIiwiY2l0YXRpb25zIjpbeyJkaXNwbGF5VGV4dCI6IklzbGFtLCBSYXNoZWR1bCBldCBhbCwgMjAyMSIsImd1aWQiOiI1NTdlNzRmZC1kZmU2LTQzZWEtOGRiYy04NzdkZjJkY2NjMDkiLCJncm91cEd1aWRzIjpbIjA1ZTYxNzhmLWEzNTUtNDA1Yy1hZGVmLTcyMzIxOWE5ZGMyNiJdLCJiaWJsaW9Db250ZW50IjpbeyJ2b2x1bWUiOiIyMiIsImRhdGUiOiIyMDIxLTA5LTAyIiwiZ3JvdXBHdWlkcyI6WyIwNWU2MTc4Zi1hMzU1LTQwNWMtYWRlZi03MjMyMTlhOWRjMjYiXSwiYXV0aG9ycyI6WyJJc2xhbSwgUmFzaGVkdWwiLCJSYWp1LCBSYWphbiBTYWhhIiwiVGFzbmltLCBOYXppYSIsIlNoaWhhYiwgSXN0aWFrIEhvc3NhaW4iLCJCaHVpeWFuLCBNYXJ1ZiBBaG1lZCIsIkFyYWYsIFl1c2hhIiwiSXNsYW0sIFRvZmF6emFsIl0sInllYXIiOiIyMDIxIiwiZWxlY3Ryb25pY1Jlc291cmNlTnVtYmVyIjoiMTAuMTA5My9iaWIvYmJhYjEwMiIsInNlY29uZGFyeVRpdGxlIjoiQnJpZWZpbmdzIGluIEJpb2luZm9ybWF0aWNzIiwiaXNibiI6IjE0NjctNTQ2MyIsImFjY2Vzc0RhdGUiOiIyMDIxLTA5LTExVDEzOjM5OjE1IiwidGl0bGUiOiJDaG9pY2Ugb2YgYXNzZW1ibGVycyBoYXMgYSBjcml0aWNhbCBpbXBhY3Qgb24gZGUgbm92byBhc3NlbWJseSBvZiBTQVJTLUNvVi0yIGdlbm9tZSBhbmQgY2hhcmFjdGVyaXppbmcgdmFyaWFudHMiLCJyZWZlcmVuY2VUeXBlIjoiMTciLCJwdWJsaXNoZXIiOiJPeGZvcmQgVW5pdmVyc2l0eSBQcmVzcyAoT1VQKSIsInJlY29yZFN0YXR1cyI6ImFjdGl2ZSIsIm51bWJlciI6IjUiLCJndWlkIjoiNTU3ZTc0ZmQtZGZlNi00M2VhLThkYmMtODc3ZGYyZGNjYzA5In1dfV19), where MEGAHIT and metaSPAdes outperformed all other assemblers.


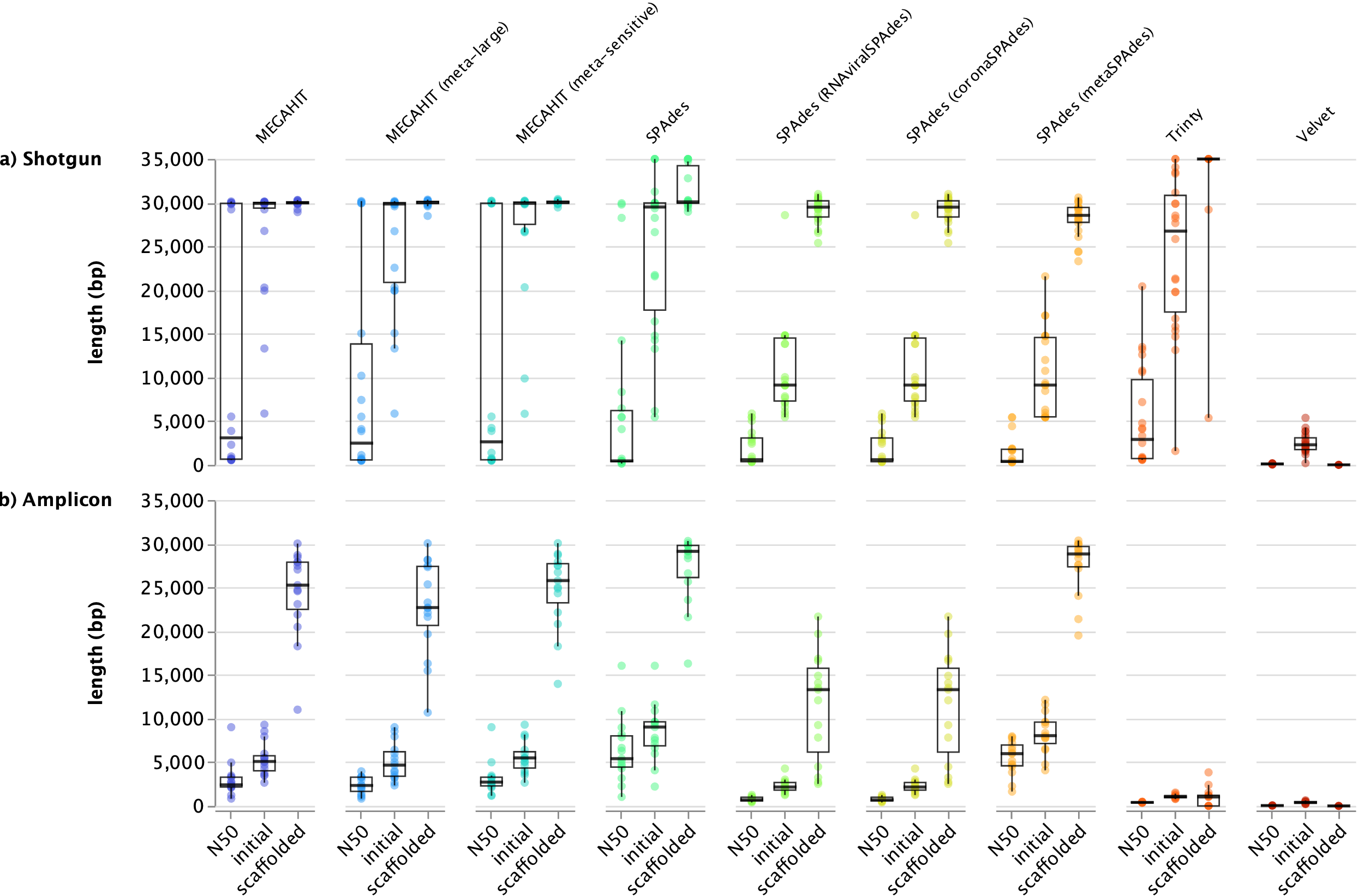


Figure A3: Comparison of MEGAHIT, SPAdes, Trinity and Velvet for SARS-CoV-2 assembly. The x-axis shows the median length (N50) from all contigs per sample, the length of the longest contig after assembly (initial) and the length of the sequence after reference-guided scaffolding (scaffolded), each in number of base pairs; a) represents patient samples from the clinical benchmark dataset, sequenced with shotgun sequencing, whereas b) the samples were sequenced with a multiplex tiling PCR (amplicon) procedure.

## Appendix 5: Specificity on Non-SARS-CoV-2 Coronaviruses

UnCoVar was tested on six non-SARS-CoV-2 coronaviruses to determine whether the workflow produced false positive results. Using mason2 [28], reads were generated based on the genome sequences of two Alphacoronaviruses and four Betacoronaviruses lineages, namely:

- human coronavirus NL63 (NCBI Reference Sequence: NC_005831.2),
- human coronavirus 229E (NCBI Reference Sequence: NC_002645.1),
- human coronavirus OC43 strain ATCC VR-759 (NCBI Reference Sequence: NC_006213.1),
- human coronavirus HKU1 (NCBI Reference Sequence: NC_006577.2),
- middle East respiratory syndrome-related coronavirus (NCBI Reference Sequence: NC_019843.3) and
- severe acute respiratory syndrome-related coronavirus Tor2 (NCBI Reference Sequence: NC_004718.3).

As expected, assembly was successful, but scaffolding did not generate consensus sequences, except for the SARS-CoV-Tor2. All viruses tested were not assigned to a SARS-CoV-2 lineage, indicating that UnCoVar does not produce false-positive results for non-SARS-CoV-2 coronaviruses.

## Appendix 6: Sequencing Depth Required for Lineage Calling

To investigate how much sequencing effort must be invested for successful lineage calling, we created several samples with varying numbers of reads (ranging from 100 to 1,000,000 reads per sample) and with three different read lengths (100 bp, 150 bp, 250 bp). For each combination, 100 samples were generated using mason2. UnCoVar was executed on these samples. Correct lineage calls obtained from preprocessed reads, the largest assembled contig, the scaffolded sequence, the variant polished, scaffolded sequence, and the pseudoassembled sequence are summarized in Figure A4.


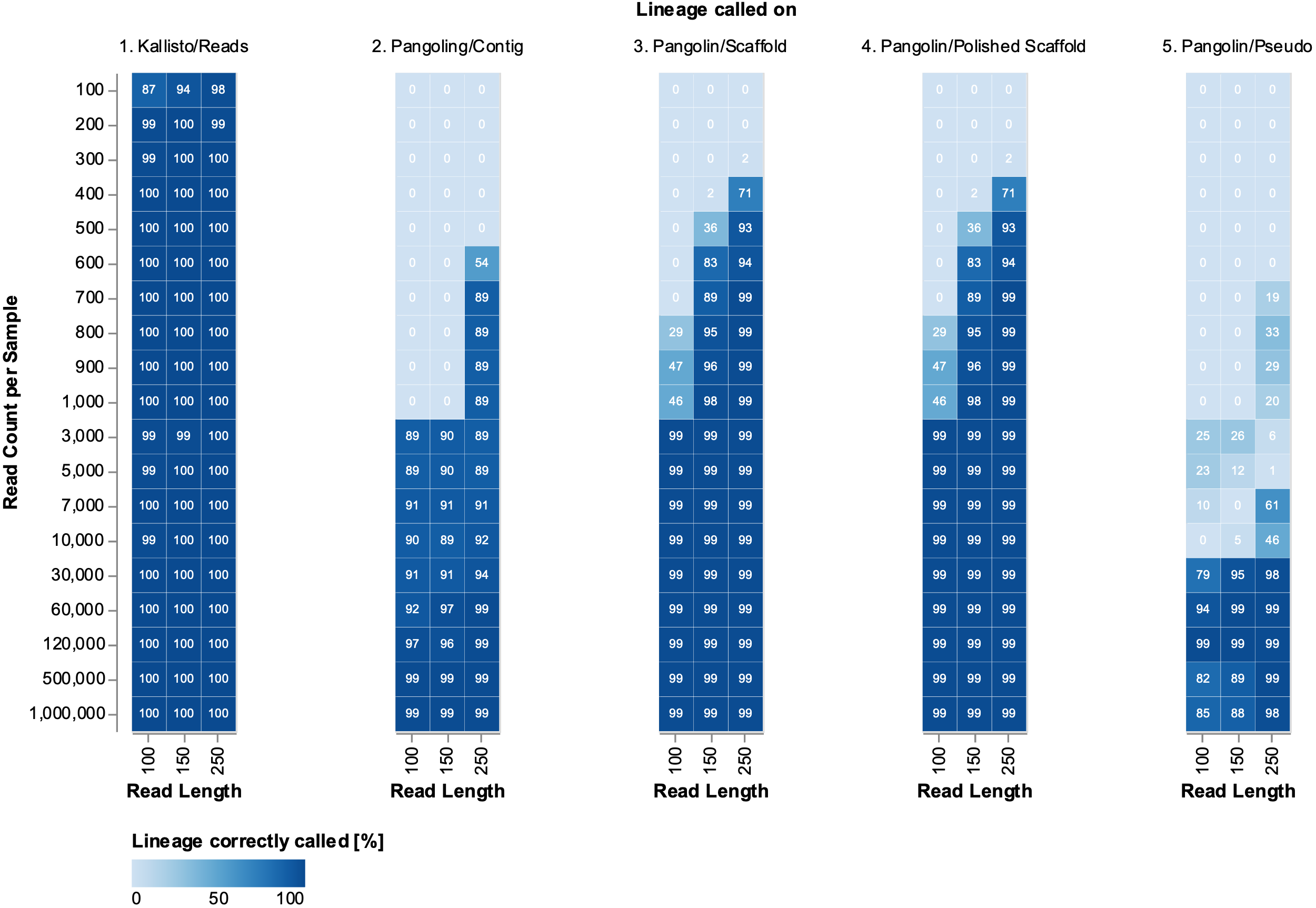


Figure A4: Number of correct lineage calls on samples with varying number and length of reads. The x-axis shows the length of the reads in the simulated samples; the y-axis shows the length of the reads in the simulated samples. The columns show the different processing states of the simulated samples across the workflow. The numbers in the boxes and their colors correspond to the correct lineage calls of the respective tools.

As can be seen in Figure A4.1, Kallisto could identify the correct lineage based on the preprocessed reads in most cases, even for samples with a low read count and length. For lineage calls that are conducted with Pangolin (Figure A4.2-4), greater sequencing effort must be made, as sufficient data must be available for good-quality assembly. It is also apparent that scaffolding after assembly is beneficial for lineage calling, as more samples could be correctly called with lower read counts and lengths. Furthermore, polishing of the scaffolded sequences did not affect the determination of lineage (Figure A4.3+4). Last, lineage calling on the pseudoassembled sequences required even more data than lineage calling on the assembly. As these results derive from artificially generated reads, the outcome could differ with the use of real data, as those can be more complex. The variant calls need a certain probability, which cannot be determined accurately with insufficient coverage of the respective base. These "weakly" represented variants are evaluated with a nonsignificant variant allele frequency and therefore are not used for constructing the reference-guided pseudoassembly. Moreover, the reference-guided pseudoassembly requires 10x coverage (this factor can be changed in the configuration file).

Based on this result, we would recommend a minimum sequencing depth of 3000 reads per sample with a read length of 100 bp, 700 to 1000 reads with a read length of 150 bp and 500 reads if you use a read length of 250 bp, to obtain a correct lineage identification based on assembled genomes. However, if that sequencing depth is not achieved, Kallisto can provide an adequate fallback, with as little as 100 reads.

##

##

## Appendix 7: Other Pipelines

Table A1: Overview of all pipelines tested for comparison, * indicates the availability of methods for processing both Illumina and Nanopore reads.

| Pipeline | Used | Variant caller version for Nanopore data | Sequencing technology |
| --- | --- | --- | --- |
| Artic | No, only reporting empty vcf-files | nanopolish | Nanopore |
|  | yes | medaka |  |
| ncov2019-artic-nf* | no, medaka minion failing | nanopolish |  |
|  | no, medaka minion failing | medaka |  |
| nf-core-viralrecon* | no, medaka minion failing | nanopolish |  |
|  | no, medaka minion failing | medaka |  |
| poreCov | no vcf-output |  |  |
| CovPipe | yes |  | Illumina |
| nf-core-viralrecon* | yes |  |  |
| ncov2019-artic-nf* | No, Nextflow preview function is deprecated |  |  |
| signal | No, fails because of Pangolin update, which uses GitHub API, which rate is limited to 60 requests |  |  |
| Snakelines | No, bowtie 2 cannot find a reference, required by the default software dependencies |  |  |
| V-pipe | yes |  |  |

## Appendix References

1. **Pathogen detection and characterization with Revelo RNA-Seq**.

2. Itokawa K, Sekizuka T, Hashino M, Tanaka R, Kuroda M: **Disentangling primer interactions improves SARS-CoV-2 genome sequencing by multiplex tiling PCR**. *PLoS One* 2020, **15**(9):e0239403.

3. **nCoV-2019 sequencing protocol V.1** [<https://www.protocols.io/view/ncov-2019-sequencing-protocol-bp2l6n26rgqe/v1?version_warning=no>]

4. Quick J, Grubaugh ND, Pullan ST, Claro IM, Smith AD, Gangavarapu K, Oliveira G, Robles-Sikisaka R, Rogers TF, Beutler NA *et al*: **Multiplex PCR method for MinION and Illumina sequencing of Zika and other virus genomes directly from clinical samples**. *Nat Protoc* 2017, **12**(6):1261-1276.

5. **nCoV-2019 sequencing protocol v3 (LoCost)** [<https://www.protocols.io/view/ncov-2019-sequencing-protocol-v3-locost-bp2l6n26rgqe/v3>]

6. Li H: **Aligning sequence reads, clone sequences and assembly contigs with BWA-MEM**. *arXiv preprint arXiv:13033997* 2013.

7. Li H: **Minimap2: pairwise alignment for nucleotide sequences**. *Bioinformatics* 2018, **34**(18):3094-3100.

8. Chen S, Zhou Y, Chen Y, Gu J: **fastp: an ultra-fast all-in-one FASTQ preprocessor**. *Bioinformatics* 2018, **34**(17):i884-i890.

9. Au CH, Ho DN, Kwong A, Chan TL, Ma ESK: **BAMClipper: removing primers from alignments to minimize false-negative mutations in amplicon next-generation sequencing**. *Scientific Reports* 2017, **7**(1):1567.

10. Li D, Liu CM, Luo R, Sadakane K, Lam TW: **MEGAHIT: an ultra-fast single-node solution for large and complex metagenomics assembly via succinct de Bruijn graph**. *Bioinformatics* 2015, **31**(10):1674-1676.

11. Nurk S, Meleshko D, Korobeynikov A, Pevzner PA: **metaSPAdes: a new versatile metagenomic assembler**. *Genome Res* 2017, **27**(5):824-834.

12. Alonge M, Soyk S, Ramakrishnan S, Wang X, Goodwin S, Sedlazeck FJ, Lippman ZB, Schatz MC: **RaGOO: fast and accurate reference-guided scaffolding of draft genomes**. *Genome Biol* 2019, **20**(1):224.

13. Garrison E, Marth G: **Haplotype-based variant detection from short-read sequencing**. *arXiv preprint arXiv:12073907* 2012.

14. Rausch T, Zichner T, Schlattl A, Stütz AM, Benes V, Korbel JO: **DELLY: structural variant discovery by integrated paired-end and split-read analysis**. *Bioinformatics* 2012, **28**(18):i333-i339.

15. Köster J, Dijkstra LJ, Marschall T, Schönhuth A: **Varlociraptor: enhancing sensitivity and controlling false discovery rate in somatic indel discovery**. *Genome Biol* 2020, **21**(1):98.

16. Danecek P, Bonfield JK, Liddle J, Marshall J, Ohan V, Pollard MO, Whitwham A, Keane T, McCarthy SA, Davies RM, Li H: **Twelve years of SAMtools and BCFtools**. *Gigascience* 2021, **10**(2).

17. O'Toole Á, Scher E, Underwood A, Jackson B, Hill V, McCrone JT, Colquhoun R, Ruis C, Abu-Dahab K, Taylor B *et al*: **Assignment of epidemiological lineages in an emerging pandemic using the pangolin tool**. *Virus Evol* 2021, **7**(2):veab064.

18. Rambaut A, Holmes EC, O'Toole Á, Hill V, McCrone JT, Ruis C, du Plessis L, Pybus OG: **A dynamic nomenclature proposal for SARS-CoV-2 lineages to assist genomic epidemiology**. *Nat Microbiol* 2020, **5**(11):1403-1407.

19. Bray NL, Pimentel H, Melsted P, Pachter L: **Near-optimal probabilistic RNA-seq quantification**. *Nature Biotechnology* 2016, **34**(5):525-527.

20. Satyanarayan A, Moritz D, Wongsuphasawat K, Heer J: **Vega-lite: A grammar of interactive graphics**. *IEEE transactions on visualization and computer graphics* 2016, **23**(1):341-350.

21. VanderPlas J, Granger B, Heer J, Moritz D, Wongsuphasawat K, Satyanarayan A, Lees E, Timofeev I, Welsh B, Sievert S: **Altair: interactive statistical visualizations for Python**. *Journal of open source software* 2018, **3**(32):1057.

22. Davis MP, van Dongen S, Abreu-Goodger C, Bartonicek N, Enright AJ: **Kraken: a set of tools for quality control and analysis of high-throughput sequence data**. *Methods* 2013, **63**(1):41-49.

23. Ewels P, Magnusson M, Lundin S, Käller M: **MultiQC: summarize analysis results for multiple tools and samples in a single report**. *Bioinformatics* 2016, **32**(19):3047-3048.

24. **FastQC: A quality control tool for high throughput sequence data.** [<https://www.bioinformatics.babraham.ac.uk/projects/fastqc/>]

25. Gurevich A, Saveliev V, Vyahhi N, Tesler G: **QUAST: quality assessment tool for genome assemblies**. *Bioinformatics* 2013, **29**(8):1072-1075.

26. Fernandes JD, Hinrichs AS, Clawson H, Gonzalez JN, Lee BT, Nassar LR, Raney BJ, Rosenbloom KR, Nerli S, Rao AA *et al*: **The UCSC SARS-CoV-2 Genome Browser**. *Nat Genet* 2020, **52**(10):991-998.

27. Mölder F, Jablonski KP, Letcher B, Hall MB, Tomkins-Tinch CH, Sochat V, Forster J, Lee S, Twardziok SO, Kanitz A *et al*: **Sustainable data analysis with Snakemake**. *F1000Research* 2021, **10**:33.

28. Holtgrewe M: **Mason – A Read Simulator for Second Generation Sequencing Data**. In*: 2010*.
